# A linamarase transgene controlled by heatshock creates a pro-toxin activation system in *Drosophila melanogaster* .

**DOI:** 10.17912/micropub.biology.001525

**Published:** 2025-04-24

**Authors:** Lauren Carey, Osnat Malka, Shai Morin, Charles Robin

**Affiliations:** 1 University of Melbourne, Melbourne, Victoria, Australia; 2 Hebrew University of Jerusalem, Israel

## Abstract

Linamarase is a plant β-glucosidase enzyme involved in the activation of plant protoxins. It thereby plays a key role in plant defense mechanisms against herbivory. We have taken the linamarase gene sequence from cassava and placed it into the genome of Drosophila melanogaster under the control of non-leaky heat-shock promoter. We show that Drosophila larvae carrying the transgene become sensitive to the pro-toxin linamarin after heat-shock. Furthermore, the sensitivity is elevated in sealed containers and control-larvae sharing such containers with linamarase-larvae are also sensitive, suggesting that the larvae are dying from poisoning with gaseous hydrogen cyanide.

**Figure 1. The linamarase gene of cassava was placed under heat shock control into the genome of Drosophila melanogaster:**
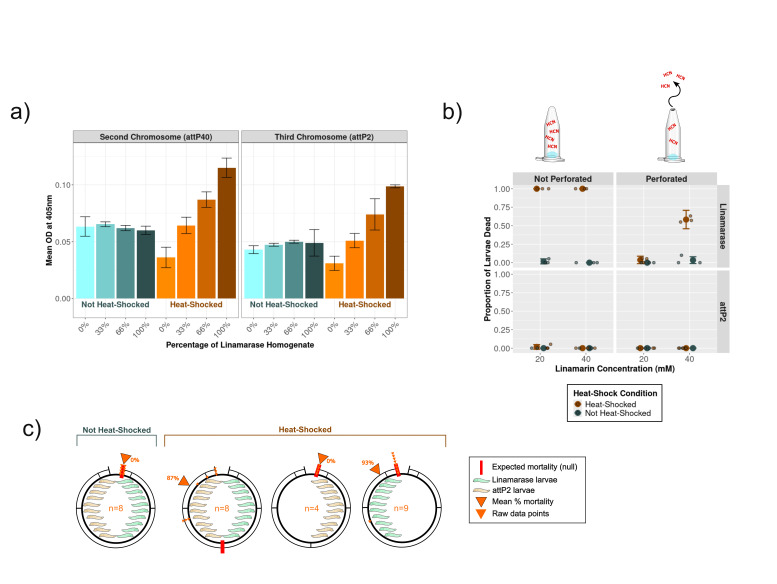
(a) p-Nitrophenol production (absorbance at an Optical Density of 405nm) from homogenates of third instar larvae after heat shock (brown tones) or without heat-shock (blue tones) with the linamarase transgene on the second or third chromosome. The larvae bearing the linamarase transgene were mixed in different percentages with attP40 or attP2 control larvae before homogenization. (b) The mortality of first instar larvae soaked in 20mM or 40mM linamarin was less when the Eppendorf tubes were perforated. There was no mortality when flies were not heat-shocked (blue) or if control flies (attP2; lower panel) were used. (c) First instar larvae bearing the control genotype (attP2) died when co-housed with first instar larvae that bore the linamarase transgene (linIII) and were heat shocked on 5mM of linamarin. The red arrows on each dial show the average % mortality in the tube whereas the solid red line shows the expectation if poisoning was limited to linamarase flies. The number of independent tubes for each treatment (n) are shown. Some images used in this figure were sourced from https://bioicons.com/.

## Description

Many plants produce two-component chemical defenses that protect them against attack from herbivorous insects (Poulton 1990). Cassava ( *Manihot esculenta* ) produces linamarin, a cyanogenic glucoside that circulates in the phloem. Upon herbivore induced damage, linamarin comes into contact with linamarase, a β-glycosidase localized in the cell wall (Mkpong et al. 1990). This enzyme breaks a β-D-glycosidic bond of linamarin to glucose and acetone cyanohydrin which then spontaneously decomposes to hydrogen cyanide (HCN). HCN is toxic to the insect because it inhibits respiration by binding to mitochondrial cytochrome oxidase (McMahon et al. 1995). The goal of our study was to introduce this pro-toxin activation system into *Drosophila melanogaster* .

The linamarase gene from cassava was engineered to be under the control of a non-leaky heat shock promoter (Akmammedov et al. 2017) and independently inserted into genomic landing sites on the second and third chromosomes of *Drosophila melanogaster* (Groth et al. 2004, Zirin et al. 2020) to create the *hs-linII* and *hs-linIII* strains. We used an artificial substrate, p-Nitrophenyl-β-D-glucopyranoside, known to be used by linamarase (Eksittikul et al. 1988), to establish that the transgenes were induced by heat shock from both landing sites. Mixing the test and control flies in defined ratios demonstrated that activity towards the artificial substrate increased proportionately only after heat-shock (Figure 1a).

In contrast to a previous study that had also placed cassava linamarase into *Drosophila melanogaster* (Goupil 2005), we sought to develop a larval assay rather than one that killed adult flies so that less linamarin was used. In an initial pilot experiment 1 ^st^ instar *hs-linII* larvae (50 per tube) were placed on cornmeal-molasses-yeast fly food laced with 2mM linamarin. When the larvae reached the 3 ^rd^ instar stage, half of the vials were heat shocked (37 ^o^ C for 45 minutes) and then returned to 25 ^o^ C, while the other half were kept at 25 ^o^ C throughout development (see methods for notes on temperature). This pilot showed that heat shocked larvae that carried 0 or 1 copy of the linamarase gene pupated at the same rates as non-heat shock controls whereas those that carried two copies (were homozygous for the transgene) had 24% fewer pupations when a heat shock was applied. This nearly-significant result (heat shock: 24, 29 pupae versus no heat shock: 32, 38 pupae; t-test, P=0.08), encouraged us to develop the following 1 ^st^ instar linamarase-dependent mortality assay.

First instar larvae were placed into sucrose droplets dosed with linamarin. While *Drosophila melanogaster* is known to be susceptible to cyanide poisoning (Broderick et al. 2009), we established that *Drosophila melanogaster* does not naturally activate the pro-toxin linamarin even when it is at a high concentration of 40mM (see attP2 controls of fig1b). We also confirmed that the PRexpress heat-shock promoter is not leaky (Akmammedov et al. 2017; see ‘not heat-shock’ controls of Figure 1b). In contrast, we observed mortality in heat-shocked flies bearing the linamarase transgene ( *hs-linIII* ) but not in heat-shocked control flies ( *attP2* ). Furthermore, this mortality was 100% on both 20mM and 40mM linamarin if the Eppendorf tubes were completely sealed. However, the mortality was substantially lowered if the Eppendorf tubes were perforated, presumably because the toxic cyanide gas dissipated.

This motivated us to ask whether control flies co-housed with the *hs-linIII* flies were at risk of cyanide poisoning. If the toxicity were limited to endogenous processes, then only the flies bearing the linamarase transgene would die. In that case, if ten *hs-linIII* transgene flies were mixed with ten control ( *attP2* ) flies then we would expect a mortality of 50%. However, Figure 1c shows that mortality on 5mM linamarin in mixed flies was significantly greater (139 out of the 160 larvae) than the number of flies bearing the linamarase transgene (80 of the 160), indicating a bystander effect (deviation from 50% assuming a normal distribution z=9.33, P<0.0001). In contrast, negative controls with ten heat shocked *attP2* larvae alone did not die and positive controls with ten heat shocked *hs-linIII* larvae all died (Figure 1c). We also note that there is a minimum number of *hs-linIII* larvae needed to achieve lethality; of the 13 tubes we set up with just five *hs-linIII* larvae in each, there was lethality in only four tubes, with only nine larvae out of the total of 65 dying.

Taken together, our findings indicate that we have managed to establish a novel heat-inducible pro-toxin activation system in *Drosophila melanogaster* . In the future, this system can be used to examine the consequences of cyanide poisoning including lifespan shortening (Goupil 2005) and to study the enzymes that insect pests use to avoid cyanide poisoning (Wybouw et al. 2014, Daborn et al. 2012, Heckel 2014, Easson et al. 2021). Importantly, our system can be generalized to serve as a scaffold for studying the molecular mechanisms behind the evolutionary arm-race between plants and herbivorous insects. This is because Drosophila can be engineered to carry transgenes that encode both (i) the activation process of many of the defense compounds that plants produce and are stored as β-glucoside conjugates that release active aglucones after sugar hydrolysis (Halkier 2006) and (ii) the mechanisms that herbivorous insects evolved to detoxify plant toxins (Heckel 2014).

## Methods

**Constructing Genotypes:** The coding sequence of linamarase from cassava ( *Manihot esculenta* LOC110619850; XM_043958354.1 ) was synthesized (as a G-block by IDT) and cloned into a *Bam* HI/ *Eco* RI digest of the PRExpress plasmid (Akmammedov et al. 2017) using Gibson Assembly. The existence of eight PRE repeats in the plasmid was confirmed prior to cloning following the *Kpn* I/ *Xho* I digest protocol recommended by Akmammedov et al. 2017. The resulting plasmid, which includes a mini-white gene was microinjected into white-eyed stocks containing the attP40 or attP2 landing sites (see Table for full genotypes) and orange-eyed progeny were used to generate homozygous stocks named *hs-linII* (linamarase in the *attP40* landing site on the second chromosome) and *hs-linIII* (linamarase in the *attP2* landing site on the third chromosome).

**Colourmetric assay:** Third instar larvae were placed in 200μL of 20% sucrose solution in Eppendorf tubes with pierced lids. Those that were heat-shocked were placed in a 37 ^o^ C water bath for one hour. Larvae were then washed with distilled H _2_ O and frozen with liquid nitrogen. Crude enzyme preparations were made by homogenizing the larvae in 500μL of buffer containing 0.1M citric acid and 0.2M Na _2_ HPO _4_ pH 7.5 by hand with a plastic pestle (Ghadamyari et al. 2010). A further 500μL of buffer was added, the sample was centrifuged at 4 ^o^ C at 13,000g for 15 minutes. The supernatant was passed through a 0.22μM filter (Millipore) and total protein concentration of each supernatant was quantified using a Bradford assay using bovine serum albumin as a standard. 100μL of standardized homogenate diluted with citric acid Na _2_ HPO _4_ buffer was added to 96 well plates. 45μL of 2mM p-Nitrophenyl-b-D-glucopyranoside (Sigma 2492-87-7) was then added to the wells and absorbance at 405nm was measured over the course of 2 hours. To account for background absorbance at 405nm homogenates of larvae carrying linamarase transgenes were mixed in fixed ratios with those of control flies ( *attP40* for *hs-linII* experiments or *attP2* for *hs-linIII* experiments) that did not.

**Toxicology:** Parents of the experimental animals were accustomed to lay-cages overnight. The lay-cages consisted of upturned 100mL polypropylene beakers with perforations that were placed on 50mm x 9 mm 4% agar plates made with ~5% dextrose, ~2.5% sucrose, 30mM NaOH and half the volume of apple juice. Eggs were collected in a four-hour window and then ~24 hours later, 10-20 first instar larvae were collected and placed into a 5μL droplet of 5% sucrose in the cap of a 0.2mL Eppendorf tube. The assay was commenced by addition of linamarin-sucrose solution to reach a final volume of 10μL and concentration of either 0.005M, 0.02M or 0.04M linamarin and 5% sucrose. After 4 hours, the larvae that were heat-shocked were placed in a water bath at 37 ^o^ C for one hour then placed in a constant temperature room for 16 to 20 hours. The constant temperature room was set to 25 ^o^ C but the sensor in that room recorded that it typically fluctuated plus and minus a degree on a daily basis. As HCN has a boiling point of 25.6 degrees and a pKa of 9.3, those repeating this protocol are advised to use a temperature of 26 ^o^ C. The sucrose solution was slightly acidic although the pH of the posterior drosophila gut can reach higher than 10 (Shanbhag and Tripathi 2009) where HCN may dissociate into CN- ions that may be less able to pass through cellular membranes.

Mortality was measured as lack of mouth-hook pumping or other body movement.

**Biosafety information:** We purchased linamarin from Sigma in lots of 50mg of lyophilized powder (catalogue number 68264) to which we added 500uL of water to generate a 0.4M master stock in a safety hood wearing gloves and safety glasses as exposure could cause skin, eye and respiratory irritation. 50mg of linamarin (molecular mass =247) could theoretically generate 5.5mg of hydrogen cyanide (molecular mass =27). This is well below acute lethal dose of HCN for humans which 0.5mg-3.5mg/kg of body weight however the Safety Data Sheet warns of harmful effects of linamarin if swallowed or inhaled. Our working stocks were ten-fold diluted (or more) and we worked in small (10uL) volumes. Linamarin is consumed by humans eating cassava and other plant products. The World Health Organization has set a safe limit of 10 ppm (10mg of total cyanide per kilogram of food such as cassava flour; Burns et al. 2012). This work was performed in a PC2 insectary and all waste and fly food was placed in a dry heat oven at 80 ^o^ C for three hours in a Dry Heat Oven (in accordance to Office of Gene Technology Regulator Guidelines for Transport, Storage and Disposal of GMOs, V1.1, 2011).

## Reagents

**Table:** 

**REAGENT TYPE**	**GENOTYPE**	**DESCRIPTION**	**AVAILABLE FROM**
plasmid:	poly-PRE-Hsp70::LacZ, Mini-white, AmpR	PRExpress (Akmammedov metal. 2017). Polycomb response elements are used to make a heatshock (hsp70). Promoter that is not leaky	Addgene #122486
Fly strain:	y[1] M{RFP[3xP3.PB] GFP[E.3xP3]=vas-int.Dm}ZH-2A w[*]\int.NLS}X; P{y[+t7.7]=CaryP}attP40	2 ^nd^ chromosome attP40 landing site.	BDSC by crossing #25709 and #24749
Fly strain:	y[1] M{RFP[3xP3.PB] GFP[E.3xP3]=vas-int.Dm}ZH-2A w[*]\int.NLS}X, y[1] sc[1] v[1] sev[21]; P{y[+t7.7]=CaryP}attP2	3 ^rd^ chromosome attP2 landing site.	BDSC by crossing #25710 and #24749
Fly strain:	y[1] M{RFP[3xP3.PB] GFP[E.3xP3]=vas-int.Dm}ZH-2A w[*]\int.NLS}X; P{PRExpress-lin}attP40	hs-linII	Robin lab
Fly strain:	y[1] M{RFP[3xP3.PB] GFP[E.3xP3]=vas-int.Dm}ZH-2A w[*]\int.NLS}X, y[1] sc[1] v[1] sev[21]; P{PRExpress-lin}attP2	hs-linIII	Robin lab
